# Supplementary material for: Integrating Individual Factors to Construct Recognition Models of Consumer Fraud Victimization
Source: Int J Environ Res Public Health. 2022 Jan 1;19(1):461. doi: 10.3390/ijerph19010461 (PMC8744553; doi:10.3390/ijerph19010461)
Supplement: Supplementary file 1 [file ijerph-19-00461-s001.zip › ijerph-1498352-supplementary.pdf]

Contents

Table S1. Feature list. 2

Table S2. Feature importance of fraud exposure recognition (FER) model. 11

Table S3. Feature importance of fraud victimhood recognition (FVR) model. 17

Table S1. Feature list.

Feature name	Feature id	Description
fraud exposure	e3016_7_mc	Is the respondent exposed to consumer fraud?
fraud victimhood	e3018	Is the respondent a fraud victim?
sex	a2003	Female or male?
age	a2005	Age
education	a2012	Educational level; from 1 (no schooling at all) to 9 (doctorate degree)
Chinese Communists	a2015	Is a Chinese Communist Party member?
registered residence	a2022	Having which type of registered residence (Hukou)? 1. Agricultural residence; 2. Non-agricultural residence; 3. Uniform registered residence; 4. Others
marital status	a2024	1. Unmarried; 2. Married; 3. Cohabitation; 4. Separated; 5. Divorced; 6. Widowed
physical condition	a2025b	Compared to peers, what's your physical condition? From 1 (very good) to 5 (very bad).
chronic disease	a2025ba	Does the respondent have a chronic disease?
employment	a3000	Does the respondent have a job?
pension	f1001a	Whether has pension?
medical insurances	f2001a	Whether has medical insurances?
housing fund	f4001	Does the respondent have a housing fund?
commercial insurance	f6001	Whether has commercial insurances?
children	a2045	Do you have any children?
financial knowledge 1	a4002a	What is your degree of concern for economic and financial information? From 1 (extremely concerned) to 5 (not at all)
financial knowledge 2	a4002b	Have you ever taken economic and financial classes?
risk appetite 1	a4003	Which of the choice below do you want to invest most if you have adequate

		money? From 1 (Project with high-risk and high-return), 2 (Project with slightly high-risk and slightly high-return) to 5 (Unwilling to carry any risk); 6. No idea
financial knowledge 3	a4004a	Given a 4% interest rate, how much would you have in total after 1 year if you have 100 yuan deposited? 1. Under 104; 2. 104; 3. Over 104; 4. Cannot figure out
financial knowledge 4	a4005a	With an interest rate of 5% and an inflation rate of 3%, the staff you buy with the money you have saved in the bank for 1 year is? 1. More than last year; 2. The same as last year; 3. Less than last year; 4. Cannot figure out
risk appetite 2	a4006a	What would you choose between a lottery with 100% shot at 4,000 yuan and another with 50% shot at 10,000 yuan and 50% chance for nothing? 1. The former 2. The latter
immigration attitude	a4010b	Do you have plans to emigrate? 1. Yes; 2. It depends; 3. No
happy	a4011c	How happy do you feel? From 1 (extremely happy) to 5 (extremely unhappy)
agricultural production	b1001	Did your family engage in agricultural production last year?
industrial and commercial operations	b2000b	Is your family engaged in production and operation of industry and commerce?
willingness to business production	b2000d	Does your household intend to participate in business production and management in the future?
housing situation	c1001	Is the house the family lives in right now is owned by family members, rented, or free? 1. Owned by family members; 2. Rented; 3. Free
outstanding loans for housing	c2024	Does the family have any outstanding loans for purchasing, decorating, remodeling, or expanding the house?
satellite receiver	c8001_10_mc	Whether own a satellite receiver?
musical instruments	c8001_12_mc	Whether own musical instruments?
cell phone	c8001_13_mc	Whether own a cell phone?
other durable goods	c8001_14_mc	Whether own other durable goods?
induction cooker	c8001_15_mc	Whether own a induction cooker?

microwave oven	c8001_16_mc	Whether own a microwave oven?
water dispenser	c8001_17_mc	Whether own a water dispenser?
durable goods	c8001_18_mc	Whether own durable goods?
camera	c8001_1_mc	Whether own a camera?
color TV	c8001_2_mc	Whether own a color TV?
washing machine	c8001_3_mc	Whether own a washing machine?
refrigerator	c8001_4_mc	Whether own a refrigerator?
air conditioner	c8001_5_mc	Whether own a air conditioner?
computer	c8001_6_mc	Whether own a computer?
stereo	c8001_7_mc	Whether own a stereo?
electric water heater	c8001_8_mc	Whether own a electric water heater?
furniture	c8001_9_mc	Whether own furniture?
demand deposit	d1101	Does your household currently have an RMB passbook or deposit card?
demand deposit amount	d1105_imp	What is the total balance of the above mentioned accounts?
fixed time deposit	d2101	Does the family currently have immature RMB fixed time deposits including certificate of time deposit?
Bank/Credit union branch counters	d2107_1_mc	Dummy variable for bank services. What major banking services do you use most frequently? (Multiple choice)
Online banking	d2107_2_mc	
Telephone banking	d2107_3_mc	
Preferential agricultural financial service points for villages	d2107_4_mc	
Mobile banking	d2107_5_mc	
Self-service internet banking	d2107_6_mc	
Other bank services	d2107_7_mc	
opinion of banking services	d2107a	What is your overall opinion of banking services? From 1 (quite satisfied) to 5 (quite dissatisfied)

stock account	d3101	Does your family have any stock accounts?
stock knowledge	d3101a	Have you heard of stocks?
fund	d5102	Does the family have any funds?
financial products of bank	d7102	Has your family had any financial products of bank now?
reason 10 for rejecting online financial products	d7106e_10_mc	Dummy variable for "why hasn’t your household purchased any internet financial products?" (Multiple choice) 1. Never heard of them; 2. High risk; 3. Low-return; 4. Don’t know how to purchase them; 5. Cumbersome purchasing procedures; 6. Lack relevant knowledge; 7. Limited funds; 8. No interest in purchasing; 9. No time or energy; 10. Having other investment channels; 11. Others
reason 11 for rejecting online financial products	d7106e_11_mc	
reason 1 for rejecting online financial products	d7106e_1_mc	
reason 2 for rejecting online financial products	d7106e_2_mc	
reason 3 for rejecting online financial products	d7106e_3_mc	
reason 4 for rejecting online financial products	d7106e_4_mc	
reason 5 for rejecting online financial products	d7106e_5_mc	
reason 6 for rejecting online financial products	d7106e_6_mc	
reason 7 for rejecting online financial products	d7106e_7_mc	
reason 8 for rejecting online financial products	d7106e_8_mc	
reason 9 for rejecting online financial products	d7106e_9_mc	
credit card	e2002	Does your family have any credit cards, excluding inactivated cards?
shop online	e2019b	Did your family shop online last month?

outstanding loans for other reasons	e3001	Has your family taken out any other loans in addition to the previously mentioned housing, automobile, commercial, educational, and credit card financing?
borrow money	e3005a	Does your household need to borrow money for housing, investing, education, or medical expenses?
outstanding medical debt	e4001	Does your household currently have any outstanding medical debt?
food expenses 1	g1001_imp	What was the average monthly amount your family spent on food last year, including dining out? (Unit: yuan)
food expenses 2	g1002_imp	What was the average amount the family spent eating out last year? (Unit: yuan)
living expenses 1	g1005_imp	What was the average monthly amount that the family spent on water, electricity, fuel, property management fees, and maintenance costs last year? (Unit: yuan)
living expenses 2	g1006_imp	What was the average monthly amount your family spent on daily necessities last year? (These include<br>items such as detergent and soap but do not include food or clothing.) (Unit: yuan)
living expenses 3	g1007_imp	What was the average monthly amount your family spent on nannies, hourly workers, drivers and other household services last year? (Unit: yuan)
transportation expenses	g1008_imp	What was the average monthly amount your family spent on local transportation last year? (Unit: yuan)
communication expenses	g1009_imp	What was the average monthly amount your family spent on communication expenses such as telephone and internet fees last year? (Unit: yuan)
mobile phone type	g1009a	Which kind of mobile phone do you use? 1. Smart phone (capable for online shopping, chatting, etc.); 2. Not a smart phone; 3. No mobile phone.
entertainment expenses	g1010_imp	What was the average monthly amount your family spent on newspapers, magazines, CDs, movie and show tickets, night clubs, cybercafés, and other education or entertainment related activities last year ? (Unit: yuan)
clothing expenses	g1011_imp	What was the average monthly amount your family spent on clothes last

		year? (Unit: yuan)
living expenses 4	g1012_imp	What was the amount your family spent on housing decoration, maintenance or expansion last year? (Unit: yuan)
heating expenses	g1013_imp	What was the amount your family spent on heating last year? (Unit: yuan)
household durable expenses	g1014_imp	What was the amount your family spent on color television sets, refrigerators, washing machines and other household durables last year? (Unit: yuan)
luxury expenses	g1015_imp	What was the amount your family spent buying luxury goods such as designer handbags or calligraphy/art and so on last year? (Unit: yuan)
education expenses	g1016_imp	What was the amount the family spent on education and training last year? (Unit: yuan) (Including job training, attending school, hobby related classes, study, health costs, etc.)
transportation purchase expenses	g1017_imp	What was the amount your family spent on purchasing cars, motorcycles, electro-mobiles, and other means of transportation last year? (Unit: yuan)
travel expenses	g1018_imp	What was your family's total expenses for traveling and visiting relatives last year? (Unit: yuan)
medical insurance expenses	g1019b_imp	How much was paid for your family's medical insurances? (Unit: yuan)
medical insurance reimbursement amount	g1019a_imp	How much was reimbursed by your family's medical insurances? (Unit: yuan)
transfer income 1	h1001	Did your family receive a gift of more than 100 yuan in cash or non-cash from persons outside the family last year?
transfer income 2_10	h2001_10_mc	dummy variable for "Did your family receive any of the following subsidies or government grants last year? (Multiple choice)" 1. None; 2. Welfare Grant; 3. One Child Incentives; 4. Five guarantee grants; 5. Pensions; 6. Relief funds; 7. Food subsidies; 8. Forestation Grants; 9. Others; 10. Subsidy for minimun living; 11. Educational subsidy; 12. Housing subsidy
transfer income 2_11	h2001_11_mc	
transfer income 2_12	h2001_12_mc	
transfer income 2_1	h2001_1_mc	
transfer income 2_2	h2001_2_mc	
transfer income 2_3	h2001_3_mc	
transfer income 2_4	h2001_4_mc	

transfer income 2_5	h2001_5_mc	
transfer income 2_6	h2001_6_mc	
transfer income 2_7	h2001_7_mc	
transfer income 2_8	h2001_8_mc	
transfer income 2_9	h2001_9_mc	
transfer income 3_1	h2003_1_mc	dummy variable for "in addition to the incomes listed above, did your family receive income in any of the following ways?" (Multiple choice) 1. Lottery; 2. Sale of a house; 3. Sale of a car; 4. Sale of intellectual property; 5. Termination indemnity; 6. Playing cards, mahjong; 7. Others; 8. None
transfer income 3_2	h2003_2_mc	
transfer income 3_3	h2003_3_mc	
transfer income 3_4	h2003_4_mc	
transfer income 3_5	h2003_5_mc	
transfer income 3_6	h2003_6_mc	
transfer income 3_7	h2003_7_mc	
transfer income 3_8	h2003_8_mc	
purpose of marriage	h3008a	What is the primary purpose of marriage? 1. Support parents; 2. Romantic life and a companion; 3. Raising a child; 4. Others
boy or girl	h3013	B oy or girl, which one is better? 1. Boy is better; 2. Girl is better; 3. The same
reason 1 for giving birth	h3014_1_mc	dummy variable for "why should someone have a child? (Multiple choice)" 1. Reproduction; 2. Love children, based on emotional consideration; 3. Raise children to take care the parents when they get old; 4. Maintain stable marriage; 5. Others
reason 2 for giving birth	h3014_2_mc	
reason 3 for giving birth	h3014_3_mc	
reason 4 for giving birth	h3014_4_mc	
reason 5 for giving birth	h3014_5_mc	
attitude towards children 1	h3015	Do you often communicate with your child? From 1 often to 5 almost never
attitude towards children 2	h3016	Could you accept your child's choice to be single? 1. Could; 2. Could not; 3. Indifferent (Do not read out)
attitude towards children 3	h3017	What would be y our attitude towards your child's choice not to have child? 1. Care; 2. Would not care; 3. Indifferent (Do not read out)
attitude towards children	h3018	Which character trait do you prefer for your child to have? 1. Obey their

4		parents, filial piety; 2. Independent thinking, having his or her own ideas
attitude towards children 5	h3019	What is the definition of filial piety? 1. Giving money to parents in their old age; 2. Caring for parents in their old age; 3. Obeying parents before turning 18
attitude towards children 6	h3020	Parents generally have the last say about important family issues regardless of the age of children, do you agree with that? 1. Totally disagree; 2. Partially disagree; 3. Don't care; 4. Partially agree; 5. Totally agree
attitude towards children 7	h3023	Children must be filial no matter what kinds of parents they are, do you agree? 1. Totally disagree; 2. Partially disagree; 3. Don't care; 4. Partially agree; 5. Totally agree
attitude towards family 1	h3024_1_mc	dummy variable for "What is the main function of a family in your opinion? (Multiple choice)" 1. Production unit; 2. Living security unit of giving birth, the old, sick, disabled; 3. Sentiment communication
attitude towards family 2	h3024_2_mc	
attitude towards family 3	h3024_3_mc	
attitude towards family 4	h3025	How important is family? 1. Very important; 2. Important; 3. General; 4. Unimportant; 5. Very unimportant
ancestor worship	h3027	Did you take part in the family's ancestor worship/tomb sweeping activities last year?
borrowing money channels	h3036	If you can borrow money from all of the following channels, which one is the most reliable way you think? 1. Bank; 2. Relatives; 3. Friends; 4. Business partners; 5. Others
trust in commercial pension insurance	h3041	If you have bought commercial pension insurance, do you believe it will repay you in the future as promised? 1. Can't believe it at all; 2. Inclined to not believe; 3. Between believe and not believe; 4. Inclined to believe; 5. Completely believe
trust in governmental pension insurance	h3042	If you have bought governmental pension insurance, do you believe it will repay you in the future as promised? 1. Can't believe it at all; 2. Inclined to not believe; 3. Between believe and not believe; 4. Inclined to believe; 5. Completely believe
old age living 1	h3044	Do you have any old age plan?

old age living 2	h3046	Who is responsible for the life of a senior citizen who has children? 1. Mainly the government; 2. Mainly the children; 3. Mainly him/herself; 4. Responsibility shared equally among the government, child, and senior
old age living 3	h3047	Which way of old age living do you choose? 1. Nursing home; 2. Living by institution; 3. Living by community
cash	k1101_imp	How much cash does your family currently have? (Unit: yuan)
lent money	k2101	Has your family lent money to “other people” which is yet to be returned ?
total assets	asset	total assets
total income	total_income_imp	total income
total debt	debt	total debt
total consumption	total_consump	total consumption
rural	rural	whether rural household?
region	region	1. East China; 2. Central China; 3. West China

Table S2. Feature importance of fraud exposure recognition (FER) model.

FER		
Feature name	Feature importance	
	M	SD
registered residence	0.0698	0.0028
total assets	0.0493	0.0028
fund	0.0407	0.0031
total consumption	0.0366	0.0010
rural	0.0350	0.0023
total income	0.0305	0.0006
stock account	0.0275	0.0029
food expenses 1	0.0270	0.0022
electric water heater	0.0255	0.0040
age	0.0243	0.0005
living expenses 1	0.0237	0.0006
entertainment expenses	0.0236	0.0012
communication expenses	0.0216	0.0005
medical insurance expenses	0.0213	0.0005
cash	0.0202	0.0003
opinion of banking services	0.0198	0.0015
living expenses 2	0.0187	0.0007
clothing expenses	0.0183	0.0005
transportation expenses	0.0181	0.0002
demand deposit amount	0.0172	0.0006
food expenses 2	0.0146	0.0006
medical insurance reimbursement amount	0.0143	0.0006
reason 1 for rejecting online financial products	0.0126	0.0021
travel expenses	0.0121	0.0009
demand deposit	0.0120	0.0018

attitude towards children 6	0.0117	0.0005
education expenses	0.0114	0.0002
total debt	0.0113	0.0003
Self-service internet banking	0.0113	0.0011
trust in commercial pension insurance	0.0105	0.0003
heating expenses	0.0101	0.0003
mobile phone type	0.0096	0.0009
purpose of marriage	0.0092	0.0003
household durable expenses	0.0082	0.0003
old age living 2	0.0081	0.0003
risk appetite 1	0.0079	0.0005
financial knowledge 1	0.0075	0.0004
camera	0.0074	0.0011
financial knowledge 3	0.0070	0.0003
transportation purchase expenses	0.0069	0.0003
financial knowledge 4	0.0065	0.0002
attitude towards children 1	0.0065	0.0003
region	0.0063	0.0001
trust in governmental pension insurance	0.0063	0.0003
attitude towards children 7	0.0063	0.0002
agricultural production	0.0061	0.0009
Bank/Credit union branch counters	0.0061	0.0005
happy	0.0060	0.0002
physical condition	0.0059	0.0003
stock knowledge	0.0054	0.0003
attitude towards children 4	0.0052	0.0003
microwave oven	0.0050	0.0005
borrowing money channels	0.0048	0.0002
attitude towards children 3	0.0045	0.0001

willingness to business production	0.0045	0.0003
attitude towards children 2	0.0042	0.0002
housing situation	0.0042	0.0003
attitude towards family 4	0.0041	0.0002
risk appetite 2	0.0040	0.0002
boy or girl	0.0038	0.0002
ancestor worship	0.0038	0.0003
attitude towards family 3	0.0037	0.0003
transfer income 1	0.0037	0.0002
Online banking	0.0036	0.0005
old age living 3	0.0035	0.0001
living expenses 4	0.0035	0.0002
shop online	0.0035	0.0003
air conditioner	0.0034	0.0003
reason 4 for giving birth	0.0033	0.0002
pension	0.0031	0.0002
chronic disease	0.0031	0.0002
attitude towards children 5	0.0030	0.0002
reason 7 for rejecting online financial products	0.0030	0.0002
old age living 1	0.0029	0.0001
reason 2 for giving birth	0.0029	0.0002
outstanding loans for housing	0.0028	0.0002
satellite receiver	0.0028	0.0002
induction cooker	0.0027	0.0001
attitude towards family 1	0.0027	0.0001
reason 6 for rejecting online financial products	0.0026	0.0001
fixed time deposit	0.0026	0.0002
children	0.0026	0.0002
water dispenser	0.0026	0.0002

sex	0.0025	0.0001
computer	0.0025	0.0005
reason 3 for giving birth	0.0025	0.0001
marital status	0.0020	0.0001
reason 8 for rejecting online financial products	0.0020	0.0001
employment	0.0020	0.0001
industrial and commercial operations	0.0019	0.0002
lent money	0.0019	0.0002
transfer income 2_1	0.0019	0.0001
refrigerator	0.0018	0.0002
commercial insurance	0.0018	0.0001
washing machine	0.0017	0.0002
cell phone	0.0016	0.0002
stereo	0.0016	0.0001
furniture	0.0015	0.0001
medical insurances	0.0015	0.0001
borrow money	0.0014	0.0001
credit card	0.0014	0.0001
Chinese Communists	0.0012	0.0001
reason 4 for rejecting online financial products	0.0011	0.0000
musical instruments	0.0011	0.0001
reason 9 for rejecting online financial products	0.0010	0.0001
reason 2 for rejecting online financial products	0.0009	0.0001
Mobile banking	0.0008	0.0001
financial knowledge 2	0.0008	0.0001
luxury expenses	0.0008	0.0001
reason 5 for giving birth	0.0007	0.0001
outstanding medical debt	0.0007	0.0001
living expenses 3	0.0007	0.0001

transfer income 2_10	0.0007	0.0001
transfer income 2_9	0.0006	0.0001
outstanding loans for other reasons	0.0005	0.0000
immigration attitude	0.0005	0.0000
color TV	0.0005	0.0000
financial products of bank	0.0005	0.0000
transfer income 2_3	0.0004	0.0001
transfer income 2_8	0.0004	0.0001
transfer income 3_8	0.0004	0.0001
Telephone banking	0.0004	0.0000
Other bank services	0.0004	0.0001
transfer income 3_6	0.0003	0.0001
transfer income 2_7	0.0003	0.0000
reason 3 for rejecting online financial products	0.0002	0.0000
reason 5 for rejecting online financial products	0.0002	0.0000
reason 11 for rejecting online financial products	0.0002	0.0000
Preferential agricultural financial service points for villages	0.0001	0.0000
other durable goods	0.0001	0.0000
transfer income 3_7	0.0001	0.0000
transfer income 2_2	0.0001	0.0000
transfer income 2_6	0.0000	0.0000
housing fund	0.0000	0.0000
transfer income 2_11	0.0000	0.0000
reason 10 for rejecting online financial products	0.0000	0.0000
education	0.0000	0.0000
transfer income 2_5	0.0000	0.0000
durable goods	0.0000	0.0000
transfer income 3_1	0.0000	0.0000
transfer income 2_12	0.0000	0.0000

	transfer income 2_4	0.0000	0.0000
	transfer income 3_2	0.0000	0.0000
	transfer income 3_3	0.0000	0.0000
	transfer income 3_4	0.0000	0.0000
	transfer income 3_5	0.0000	0.0000

Table S3. Feature importance of fraud victimhood recognition (FVR) model.

FVR		
Feature name	Feature importance	
	M	SD
total income	0.0734	0.0117
age	0.0633	0.0100
clothing expenses	0.0482	0.0092
total assets	0.0446	0.0057
cash	0.0439	0.0061
total consumption	0.0367	0.0020
medical insurance expenses	0.0328	0.0033
total debt	0.0324	0.0046
entertainment expenses	0.0280	0.0037
living expenses 1	0.0273	0.0044
living expenses 2	0.0262	0.0057
demand deposit amount	0.0249	0.0022
communication expenses	0.0249	0.0050
transportation expenses	0.0182	0.0032
food expenses 1	0.0173	0.0022
heating expenses	0.0157	0.0024
transportation purchase expenses	0.0151	0.0045
household durable expenses	0.0148	0.0037
food expenses 2	0.0140	0.0030
medical insurance reimbursement amount	0.0137	0.0017
lent money	0.0133	0.0036
attitude towards children 1	0.0122	0.0037
happy	0.0120	0.0028
willingness to business production	0.0117	0.0028
attitude towards children 6	0.0114	0.0020

education expenses	0.0097	0.0018
borrow money	0.0094	0.0034
trust in commercial pension insurance	0.0091	0.0014
trust in governmental pension insurance	0.0090	0.0020
attitude towards children 7	0.0081	0.0016
registered residence	0.0080	0.0019
travel expenses	0.0080	0.0016
old age living 2	0.0076	0.0014
risk appetite 1	0.0074	0.0016
purpose of marriage	0.0074	0.0020
financial knowledge 1	0.0073	0.0022
physical condition	0.0070	0.0014
borrowing money channels	0.0066	0.0016
opinion of banking services	0.0066	0.0012
financial knowledge 3	0.0061	0.0012
attitude towards children 4	0.0059	0.0027
attitude towards children 3	0.0057	0.0011
financial knowledge 2	0.0056	0.0012
financial knowledge 4	0.0055	0.0013
region	0.0055	0.0016
attitude towards children 2	0.0051	0.0017
living expenses 4	0.0050	0.0023
induction cooker	0.0050	0.0016
reason 2 for giving birth	0.0049	0.0018
boy or girl	0.0046	0.0010
satellite receiver	0.0045	0.0021
old age living 1	0.0044	0.0010
air conditioner	0.0042	0.0025
commercial insurance	0.0041	0.0020

outstanding loans for housing	0.0041	0.0015
transfer income 1	0.0040	0.0022
Self-service internet banking	0.0039	0.0016
attitude towards family 3	0.0038	0.0014
chronic disease	0.0038	0.0015
microwave oven	0.0038	0.0017
attitude towards family 4	0.0037	0.0012
reason 4 for giving birth	0.0036	0.0010
Online banking	0.0035	0.0013
fund	0.0035	0.0014
agricultural production	0.0033	0.0011
Mobile banking	0.0032	0.0021
sex	0.0031	0.0010
risk appetite 2	0.0031	0.0010
electric water heater	0.0031	0.0014
rural	0.0031	0.0008
water dispenser	0.0031	0.0013
fixed time deposit	0.0031	0.0014
reason 7 for rejecting online financial products	0.0029	0.0012
stock knowledge	0.0029	0.0009
stock account	0.0029	0.0009
attitude towards family 1	0.0028	0.0012
old age living 3	0.0027	0.0012
stereo	0.0027	0.0013
mobile phone type	0.0027	0.0009
computer	0.0027	0.0008
reason 8 for rejecting online financial products	0.0026	0.0015
pension	0.0025	0.0011
employment	0.0023	0.0007

housing situation	0.0023	0.0010
industrial and commercial operations	0.0022	0.0008
ancestor worship	0.0022	0.0012
reason 3 for giving birth	0.0021	0.0010
marital status	0.0020	0.0011
transfer income 2_1	0.0020	0.0015
children	0.0020	0.0008
reason 6 for rejecting online financial products	0.0019	0.0009
shop online	0.0019	0.0013
medical insurances	0.0018	0.0015
outstanding loans for other reasons	0.0018	0.0015
Bank/Credit union branch counters	0.0016	0.0008
reason 1 for rejecting online financial products	0.0016	0.0007
attitude towards children 5	0.0015	0.0009
camera	0.0014	0.0006
transfer income 3_6	0.0013	0.0009
furniture	0.0013	0.0009
outstanding medical debt	0.0013	0.0010
credit card	0.0011	0.0008
washing machine	0.0011	0.0008
refrigerator	0.0009	0.0005
transfer income 2_8	0.0008	0.0007
luxury expenses	0.0008	0.0007
living expenses 3	0.0008	0.0007
reason 5 for giving birth	0.0008	0.0005
demand deposit	0.0007	0.0005
reason 4 for rejecting online financial products	0.0007	0.0006
immigration attitude	0.0006	0.0004
musical instruments	0.0006	0.0004

transfer income 3_8	0.0005	0.0005
reason 2 for rejecting online financial products	0.0005	0.0003
cell phone	0.0004	0.0003
transfer income 2_9	0.0003	0.0003
transfer income 2_10	0.0002	0.0002
Telephone banking	0.0002	0.0004
Other bank services	0.0002	0.0003
color TV	0.0002	0.0003
financial products of bank	0.0002	0.0003
reason 5 for rejecting online financial products	0.0001	0.0002
reason 9 for rejecting online financial products	0.0001	0.0003
Chinese Communists	0.0000	0.0001
education	0.0000	0.0000
housing fund	0.0000	0.0000
other durable goods	0.0000	0.0000
durable goods	0.0000	0.0000
Preferential agricultural financial service points for villages	0.0000	0.0000
reason 10 for rejecting online financial products	0.0000	0.0000
reason 11 for rejecting online financial products	0.0000	0.0000
reason 3 for rejecting online financial products	0.0000	0.0000
transfer income 2_11	0.0000	0.0000
transfer income 2_12	0.0000	0.0000
transfer income 2_2	0.0000	0.0000
transfer income 2_3	0.0000	0.0000
transfer income 2_4	0.0000	0.0000
transfer income 2_5	0.0000	0.0000
transfer income 2_6	0.0000	0.0000
transfer income 2_7	0.0000	0.0000
transfer income 3_1	0.0000	0.0000

	transfer income 3_2	0.0000	0.0000
	transfer income 3_3	0.0000	0.0000
	transfer income 3_4	0.0000	0.0000
	transfer income 3_5	0.0000	0.0000
	transfer income 3_7	0.0000	0.0000
